# Sonographic and pathological features of metaplastic squamous cell carcinoma of the breast: a case series

**DOI:** 10.1186/s12893-021-01375-0

**Published:** 2021-10-23

**Authors:** Xiao-Jian Ye, Xiao-Yu Chen, Zhi-Bin Ke, Fei Lin, Yu-Peng Wu, Lei Yan

**Affiliations:** 1 Department of Ultrasound, The First Affiliated Hospital of Fujian Medical University, 20 Chazhong Road, Fuzhou, 350005 China; 2 Department of Urology, Urology Research Institute, The First Affiliated Hospital, Fujian Medical University, Fuzhou, 350005 China

**Keywords:** Sonography, Breast cancer, Metaplastic squamous cell carcinoma, Pathology

## Abstract

**Objective:**

The aim of this study was to evaluate the sonographic features and to compare the sonographic findings with the pathologic features.

**Methods:**

The sonographic and pathological features of all patients were retrospectively reviewed.

**Results:**

All these 9 patients presented with a palpable breast mass first found by the patient before presentation. The median diameters were 3.67 cm. On two-dimensional imaging, 8 masses showed mixed echogenicity with both solid and cystic components, and only 1 mass showed hypoechoic. All the masses had irregular shapes. 1 mass had indistinct margin and 8 masses had microlobulated margins. Calcifications was seen in 1 mass. On color Doppler flow imaging, 8 masses had high vascularity with high resistance index; 5 masses had grade III blood flow signal; 3 masses had grade II blood flow signal. On histopathological examination, 5 masses were adenocarcinoma with squamous metaplasia, and 4 masses were pure SCC. On immunohistochemical staining, estrogen receptors (ER), progesterone receptors (PR) and human epidermal growth factor receptor (HER2) were negative in 5 masses. There were 2 patients with lymph node metastasis.

**Conclusions:**

Most of the sonographic features of MSCC were mixed echogenicity with central cystic components, posterior echo enhancement, abundant vascularity with high resistance.

## Introduction

Metaplastic squamous cell carcinoma (MSCC) is a rare subtype of metaplastic breast carcinoma (MBC). It is reported that the prognosis of MSCC is worse than breast invasive ductal carcinoma (IDC) because conventional chemotherapy for breast IDC had no effects on MSCC [1–4]. Previous reports of primary squamous cell carcinoma (SCC) or metaplastic carcinoma were limited, and most of them focused on the pathologic and clinical aspects. However, the sonographic features of MSCC were rarely reported. The aim of this study was to evaluate the sonographic features in 9 patients with breast MSCC and compare the sonographic findings with the pathologic features.

## Materials and methods

This study was approved by the Ethics Committee of the First Affiliated Hospital of Fujian Medical University and written informed consent was provide by all included patients. A total of 9 (0.26%) patients pathologically confirmed as MSCC among 3420 patients with breast malignancy in the First Affiliated Hospital of Fujian Medical University from January 2006 to January 2016 were included in this study. All methods were performed in accordance with the relevant guidelines and regulations.

Preoperative sonography of the breast and axillae was performed using high resolution linear transducers (10–18 MHz) of Philips IU22 or Acuson Sequoia 512. The sonographic images were analyzed retrospectively by two experienced sonologist. The two-dimensional sonographic findings including the lesion size, location, shape, margin, echogenicity and calcification were observed and described according to the American College of Radiology Breast Imaging Reporting and Data System (ACR BI-RADS)—Ultrasound, First Edition, 2013 [5]. The echogenicity was classified as cystic, hypoechoic, isoechoic, hyperechoic and mixed echoic. When a mass showed echogenicity minimally less than that of subcutaneous fat, it was defined as hypoechoic. When a mass showed no echo (cystic) and echo (solid) component, it was defined as mixed echo or cystic-solid mixed echo. Color Doppler ultrasound was performed to observe the distribution of blood flow, and the grades of the blood flow signal in the lesions were classified according to the Adler semi-quantitative method [6].

Gross and microscopic slides of surgical specimens were reviewed by two experienced pathologists who had expertise in breast pathology. Breast MSCC was diagnosed according to the pathological criteria [7]: MSCC was diagnosed when more than 50% of the malignant cells showed a squamous component, while pure squamous cell carcinoma (pure SCC) was diagnosed when 100% of the malignant cells showed a squamous component. The gross pathologic size and cellularity of the tumor, the amount of cyst or necrosis in the tumor, the presence of calcifications, and each patient’s lymph node status were evaluated as far as possible. The markers of estrogen receptor (ER), progesterone receptor (PR), and human epidermal growth factor receptor (HER2) were also tested by immunohistochemistry according to the following criteria [6]: 10% of tumor cells with positive nuclear staining were regarded as ER/PR positive. HER2 scoring was performed according to the manufacturer’s instructions (Hercep test; Dako, Carpinteria, CA, USA), and a score of 3+ was considered positive.

Sonographic findings and pathologic features were contrastly analyzed.

## Results

The 9 patients were all females and presented with a palpable breast mass first found by the patient before presentation. The median age was 45 years (range, 34–73 years). The median diameters of the masses were 3.67 cm (range, 2.35–5.1 cm).

**Fig. 1:**
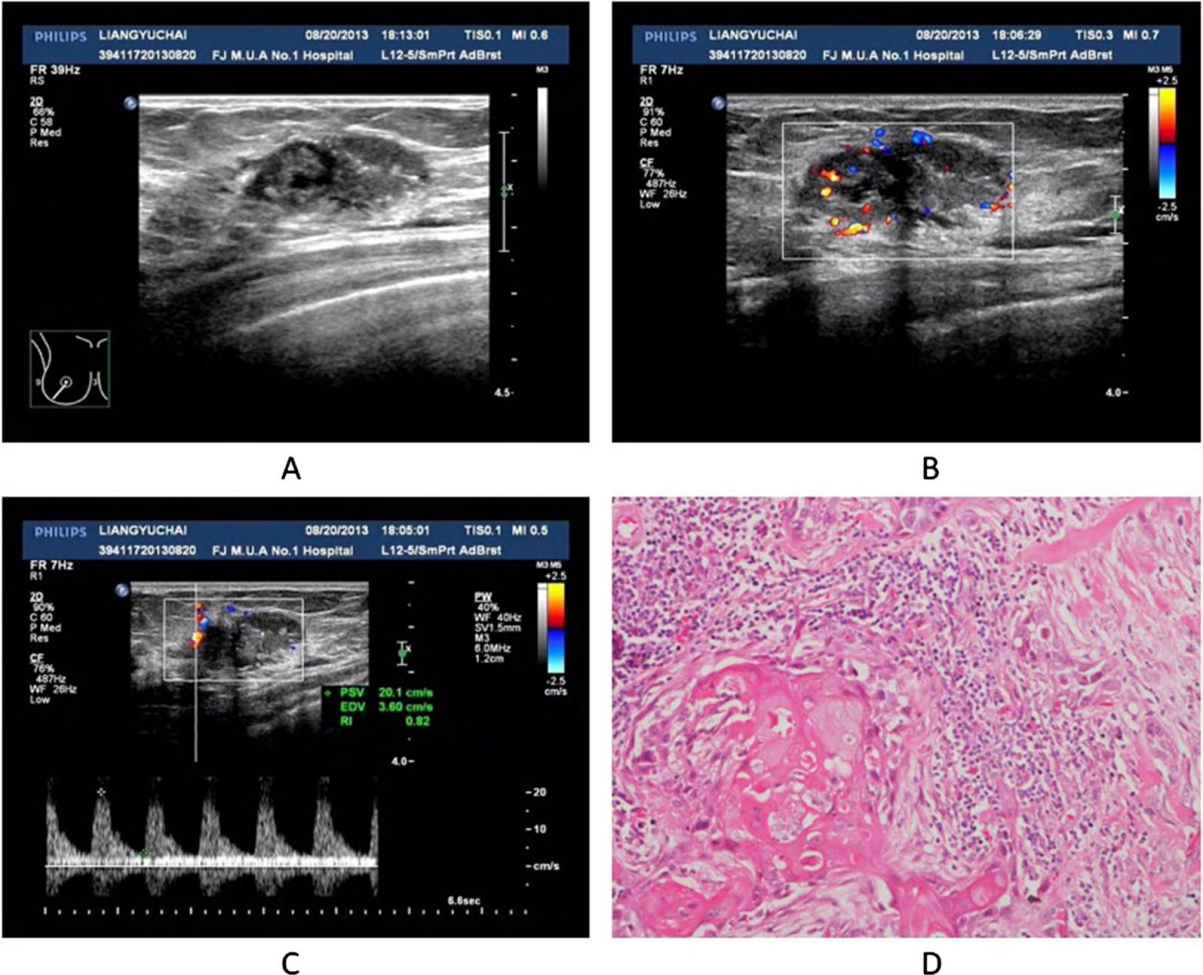
Adenosquamous carcinoma of MSCC. (Proportion of squamous cell carcinoma and invasive ductal carcinoma were 70% and 30%, respectively). **A** Two-dimensional ultrasonography (US) revealed a mixed echoic mass with an irregular shape (small cystic areas, and posterior enhancement. **B** Color Doppler ultrasound showed increased color flow signals in the solid part of the mass. **C** Spectral Doppler ultrasound showed a high resistive index in the feeding artery. **D** Histopathological examination revealed squamous epithelial differentiation and keratinization in the center of the tumor (hematoxylin–eosin, × 200)

**Fig. 2:**
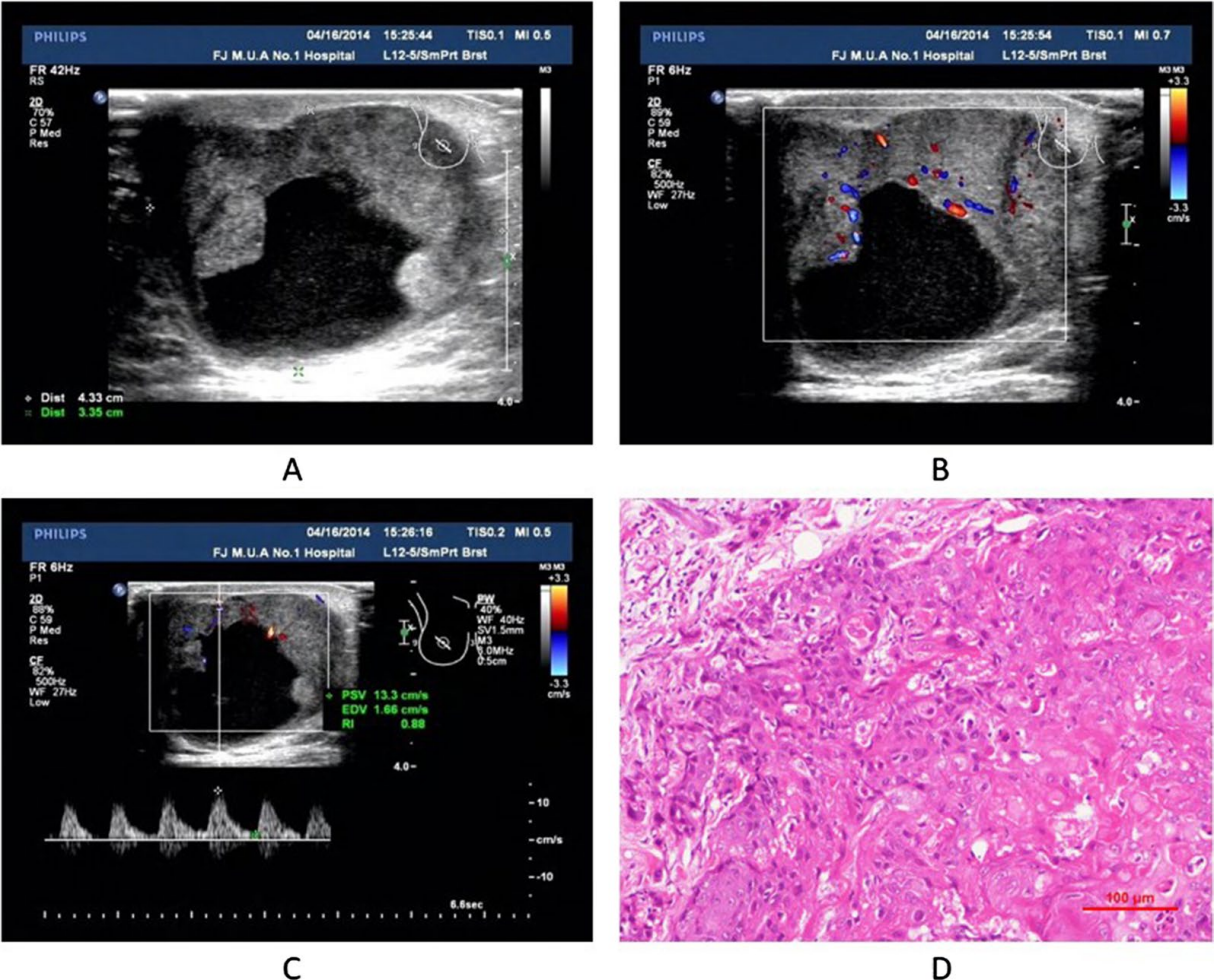
Pure squamous cell carcinoma (Pure SCC). (Proportion of squamous cell carcinoma was 100%). **A** Two-dimensional ultrasonography (US) revealed a mixed echoic mass with an irregular shape (large cystic areas, and posterior enhancement. **B** Color Doppler ultrasound showed increased color flow signals in the solid part of the mass. **C** Spectral Doppler ultrasound showed a high resistive index in the feeding artery. **D** Histopathological examination revealed squamous epithelial differentiation and keratinization in the tumor (hematoxylin–eosin, × 100)

Tables 1 and 2 lists US findings for the 9 masses. Two-dimensional ultrasonography was performed for all masses. On two-dimensional imaging, 8 (8/9, 88.89%) masses showed mixed echo with solid and cystic components (Figs. 1A and 2A), and only 1 (1/9, 11.11%) mass showed hypoechoic. All the masses in our study had irregular shape and posterior enhancement. Only 1 (1/9, 11.11%) mass had indistinct margin and 8(8/9, 88.89%) masses had microlobulated margin. Calcifications were seen in 1 (1/9, 11.11%) mass. Color and Spectral Doppler ultrasound were performed for 8 masses, and only 1 mass was not available. On color and spectral Doppler flow imaging, 8 (8/9, 88.89%) masses had rich blood flow signals (Figs. 1B and 2B) with high resistance (RI, 0.77–0.91, Figs. 1C and 2C); 5 (5/8, 62.50%) masses had III grade blood flow signals; and 2 masses (2/9, 22.22%) had II grade blood flow signals.

**Table 1:** Sonographic and pathologic findings in 9 patients with metaplastic squamous cell carcinoma

Case	Age (y)	Location	Sonographic findings								Pathologic findings			
			Shape	Margin	Size (cm)	Echo	Cal	PE	BF	RI	Subtype	SCC_percentage_	Lym	ER/PR/HER2
1	60	Right	Irregular	Microlobulated	2.61	Mixed	+	+	III	0.82	ASC	70	−	−/−/−
2	36	Left	Irregular	Indistinct	5.1	Mixed	−	+	II	0.78	ASC	75	+	+/−/−
3	34	Left	Irregular	Microlobulated	4.7	Mixed	−	+	III	0.77	ASC	75	+	+/−/−
4	44	Left	Irregular	Microlobulated	2.4	Mixed	−	+	II	0.79	ASC	90	−	−/+/−
5	41	Right	Irregular	Microlobulated	3.67	Mixed	−	+	III	0.81	ASC	95	−	−/−/+
6	73	Right	Irregular	Microlobulated	5.07	Mixed	−	+	III	0.88	SCC	100	−	−/−/−
7	56	Left	Irregular	Microlobulated	3.26	Mixed	−	+	NA	NA	SCC	100	−	−/−/−
8	45	Right	Irregular	Microlobulated	2.35	Hypoechoic	−	+	II	0.77	SCC	100	−	−/−/−
9	49	Right	Irregular	Microlobulated	4.61	Mixed	−	+	III	0.97	SCC	100	−	−/−/−

*BF* blood flow, *Cal* calcification, *HE* hypoechoic, *Lym* lymph node, *NA* not available, *PE* posterior enhancement, *RI* resistive index

**Table 2:** Sonographic and pathological characteristics of patients with MSCC

Characteristics	n	%
Sex		
Male	0	0
Female	9	100%
Age (years)		
Median (range)	45	34–73
≤ 40	2	22.22%
> 40	7	77.78%
Location		
Left	4	44.44%
Right	5	55.56%
Size (cm), (n = 9)		
Median (range)	3.67	2.35–5.1
< 5	7	77.78%
≥ 5	2	22.22%
Shape		
Irregular	9	100%
Margin		
Microlobulated	8	88.89%
Indinct	1	11.11%
Posterior enhancement	9	100%
Echo pattern		
Mixed echo	8	88.89%
Hypoechoic	1	11.11%
Calcification	1	11.11%
Axillary lymph nodes	2	22.22%
Blood flow grade		
III grade	5	55.56%
II grade	3	33.33%
NA	1	11.11%
Subtype of MSCC		
ASC	5	55.56%
Pure SCC	4	22.22%
ER, PR, HER2(−/−/−)	5	55.56%
ASC	1	11.11%
Pure SCC	4	44.44%

Tables 1 and 2 showed pathological findings for the 9 masses. Gross pathology and histopathology were performed for all masses. On gross pathological examination, 8 gross specimens showed irregular tumors with cyst necrosis and hemorrhage; the cut surface of the tumors revealed cystic lesions with central necrosis (8/9, 88.89%) or solid (1/9, 11.11%). Calcifications were found in 1 (1/9, 11.11%) mass. 2 (2/9, 22.22%) patients had ipsilateral axillary lymph node involvement. On histopathological examination, 5 (5/9, 55.56%) masses were adenosquamous carcinoma (ASC, Figs. 1D and 2D), and 4 (4/9, 44.44%) masses were pure SCC. ER was positive in 2 (2/9, 22.22%) masses and PR was positive in 1 (1/9, 11.11%) mass according to immunohistochemical staining. ER, PR and HER2 were all negative in 5 (5/9, 55.56%) masses (triple-negative breast cancer). Moreover, 4 masses of the above 5 triple-negative breast cancers were pure SCC.

## Discussion

Breast MSCC is a rare MBC [8, 9] accounting for less than 1% of patients with breast cancer [8]. Breast MSCC is a carcinoma with squamous metaplasia of adenocarcinoma arising from the breast ductal epithelium. According to the cancer registered in our hospital, only approximately 0.23% of breast malignancies were MSCC. Breast MSCC also may manifest in a pure form. In our study, pure SCC accounted for 0.10% in all cases of breast carcinoma, which was consistent with the result of previous literature [10]. The pathological subtypes of breast MSCC in our study included ASC (5/9, 55.56%) and pure SCC (4/9, 44.44%).

Breast MSCC is not usually associated with hormone receptors. Immunohistochemical studies have found that fewer than 20% of cases are hormone receptor-positive [11–13]. In the present study, ER was positive in 1 (1/9, 11.11%) patient; PR was positive in 2 (2/9, 22.22%) patients; ER, PR and HER2 were negative in 5 masses (5/9, 55.56%, triple-negative breast cancer); 4 patients of the above triple-negative breast cancer were pure SCCs. Grenier et al. [8] have described common immunohistochemical findings of pure SCC as p63 positivity, CK 5/6 positivity, and ER, PR, and HER2 negativity. However, Tse et al. [14] indicated that only a small percentage of pure SCCs have ER positivity. There was no ER positivity in pure SCCs in our study. Many tumors were triple-negative, which has been reported to indicate a poor prognosis [15].

Previous studies [16–19] have showed that breast MSCC was characterized by rapid growth. Sakurai et al. [16] revealed that the size of breast MSCCs was frequently large when they were detected. According to recent studies, about 30% of breast SCCs patients have tumor larger than 5 cm in diameter [1, 11, 20]. However, in this study, there were only 2 (2/9, 22.22%) patients with breast MSCCs larger than 5 cm in diameter, and the median tumor diameter was only 3.67 cm (range, 2.35–5.1 cm). The possible reason might be the improvement of women’s awareness of breast self-examination and the popularization of routine color Doppler ultrasound screening method.

To our knowledge, the sonographic appearance of MSCC had been rare described in previous studies. In our study, all masses were irregular in shape; the margins of masses were either indistinct (1/9, 11.11%) or microlobulated (8/9, 88.89%); only 1 patients had calcifications; all masses showed posterior acoustic enhancement; only 1 mass had hypoechogenicity and the other 8 tumors in our study had mixed echo with solid and cystic components; 8 tumors (8/9, 88.89%) showed the presence of central cystic areas of varying size on sonography. Previous studies have showed that there were approximately 60% to 80% of breast MSCCs containing a cystic component or central necrosis [12, 21, 22]. In the present study, the cystic component was identified using preoperative ultrasonography. Moreover, histopathological examination revealed a cystic lesion that correlated with necrosis, hemorrhage and cystic degeneration, which concur with MBC [23–26] on pathologic examination. The cause of internal cystic necrosis was the fast growth of squamous cell carcinoma, which easily lead to internal blood-supply insufficiency. Under the microscope, the cystic areas were squamous cell carcinoma aggregation center. In addition, we speculated that the central cystic region size might be related to the content of squamous cell carcinoma and the size of tumor. However, since breast MSCC is rare, the sample size of this study was large enough for statistical analysis. On sonographic appearance, there were some high echo deposition in the cystic areas due to falling off easily of cystic wall containing squamous cell. This kind of squamous cell aggregation necrotic cavity was particularly common in the lesions larger than 2 cm [27]. Only 1 tumor showed relative hypoechogenicity, possibly due to hypercellularity in the solid areas according to histological examination [28]. In the present study, posterior acoustic enhancement was found in all lesions, possibly due to cystic areas and/or hypercellularity. The sonographic feature of posterior acoustic enhancement in the breast MSCCs was different from posterior echo attenuation in most of the non-special types of invasive breast cancers.

On color Doppler flow imaging, only 1 tumor was not available of color and spectral Doppler. The remaining 8 (8/9, 88.89%) tumors had rich blood flow signals with high resistance (II or III grade, RI, 0.77–0.91). This was likely to be closely related to the tumor cell density and the abundant neovascularization in the carcinoma nest. RI values were 0.62 ± 0.095 in benign tumors [29] and 0.75 ± 0.07 in malignant tumors, respectively [30]. Although there is some controversy [31], the RI of malignant tumors is considered to be significantly higher than that of benign tumors. In the present study, the average RI value was 0.82, which was higher than that of benign tumors and the non-special types of invasive breast cancers. The high RI may be due to high tumor stiffness.

There were 2 (2/9, 22.22%) patients with ipsilateral axillary lymphatic nodal metastasis indicated by ultrasound in this study. This incidence was similar to that in other studies (12.5–40%) [23, 32]. It was reported that squamous cell carcinomas were less likely to have lymphatic spread than adenocarcinomas [1, 10]. Moreover, no metastatic cancer cells were found in the swollen lymph nodes in the axillary lymphatic nodal. Breast MSCCs without lymph node metastasis have a relatively good prognosis. The larger the mass, the more likely it is to metastasize [33]. In our study, the tumor size of 2 patients with lymph node metastasis were 5.1 cm and 4.7 cm, respectively. This result was consistent with previous studies. No patient developed distant metastasis in the present study. Some studies have reported a 5-year survival rate of 40% [34]. The prognostic outcome was significantly associated with the size of the mass and the presence of lymph node involvement at diagnosis [33, 34].

However, this study is a retrospective cases series in a single-center with limited sample size. The small sample size is the major limitation of our study. Due to the rare incidence of metaplastic squamous cell carcinoma of the breast, we could merely include nine patients in this study. External validation of our results with larger sample size is needed to evaluate further in future.

## Conclusions

In conclusion, breast MSCC is an unusual breast malignancy. Sonographic features for breast MSCC include relatively large size, microlobulated, mixed echo with a central cystic area, posterior acoustic enhancement, hypervascularity and high resistance index. Although breast MSCC patients may not have all of these sonographic features, MSCC should be considered in the differential diagnosis of breast malignant tumors when the above described ultrasound manifestations were encountered.

## Data Availability

All data generated or analyzed during the present study are included in this article.
